# Undervirilized male infant with in utero exposure to maternal use of high dose antifungal therapy

**DOI:** 10.1186/s13633-020-00087-1

**Published:** 2020-09-09

**Authors:** Jasmine Gujral, Gertrude Costin, Divya Khurana, Mabel Yau, Elizabeth Wallach, Christopher J. Romero, Meredith Wilkes, Swathi Sethuram, Robert Rapaport

**Affiliations:** 1 Division of Pediatric Endocrinology and Diabetes, Yale School of Medicine, New Haven, CT USA; 2 Division of Pediatric Endocrinology and Diabetes, Kravis Children’s Hospital, Icahn School of Medicine at Mount Sinai, New York, NY USA; 3 Division of Pediatric Endocrinology, The Texas Tech University Health Sciences Center, Lubbock, TX USA

**Keywords:** Adrenal insufficiency, Atypical genitalia, Endocrine disruptors, Undervirilization

## Abstract

**Background:**

Antifungals act on fungal sterols structurally similar to human cholesterol. Ketoconazole reversibly suppresses steroidogenesis by inhibiting cytochrome P450 enzymes and interferes with dihydrotestosterone (DHT) activity by binding to the androgen receptor. Hypospadias was reported in infants exposed to nystatin in utero.

**Case presentation:**

A male infant exposed to antepartum nystatin presented with severe under-undervirilization and transient adrenal corticosteroid abnormalities. He was born in USA at 31 weeks gestation to a mother treated with vaginal Polygynax capsules (nystatin-100,000 international units, neomycin sulphate-35,000 international units and polymyxin B-35,000 international units) for vaginal discharge in the Ivory Coast. She used approximately 60 capsules between the first trimester until delivery. The infant was born with micropenis, chordee, perineo-scrotal hypospadias and bifid scrotum with bilaterally palpable gonads. The karyotype was 46,XY. No Mullerian structures were seen on ultrasound. Serum 17-hydroxyprogesterone (17 OHP) on newborn screening was high (304 ng/ml, normal < 35). Cortisol response to cosyntropin on the 3rd day of life (DOL) was 10 mcg/ml; the subnormal cortisol response may have resulted from prematurity and the predelivery treatment with betamethasone. The elevation of several adrenal corticosteroids was not consistent with any specific enzymatic defect. Hydrocortisone and fludrocortisone were initiated at another hospital for suspected mild glucocorticoid and mineralocorticoid deficiencies. Genetic screening for adrenal and gonadal developmental defects performed when transferred to our care were normal. All medications were gradually discontinued over 5–8 months. Adrenal and testicular responses to cosyntropin and human chorionic gonadotropin (hCG) were normal at 8 months.

**Conclusions:**

We report severe undervirilization in a 46,XY infant born to a mother treated with prolonged and high dose nystatin during pregnancy. This presentation suggests that prolonged antepartum use of high dose nystatin could lead to severe but transient defects in androgen synthesis and/or action possibly by acting as an endocrine disruptor. Further studies are warranted to confirm this finding. Thus, endocrine disruptors should be considered in male newborns with atypical genitalia not explained by common pathologies.

## Background

Ketoconazole is a recognized endocrine disruptor that causes reversible suppression of adrenal and gonadal steroidogenesis and displaces DHT from its receptor [1]. Nystatin, presently not recognized as an endocrine disruptor, has been associated with hypospadias in males exposed to it in utero [2].

We present a severely undervirilized male infant with in utero exposure to high dose nystatin from the 8th week of gestation until delivery. Neonatal hormonal assessment revealed multiple adrenal corticosteroid abnormalities for which he was treated. All hormonal abnormalities were transient and resolved by 8 months of age. No genetic defect was demonstrated.

## Case presentation

**Fig. 1:**
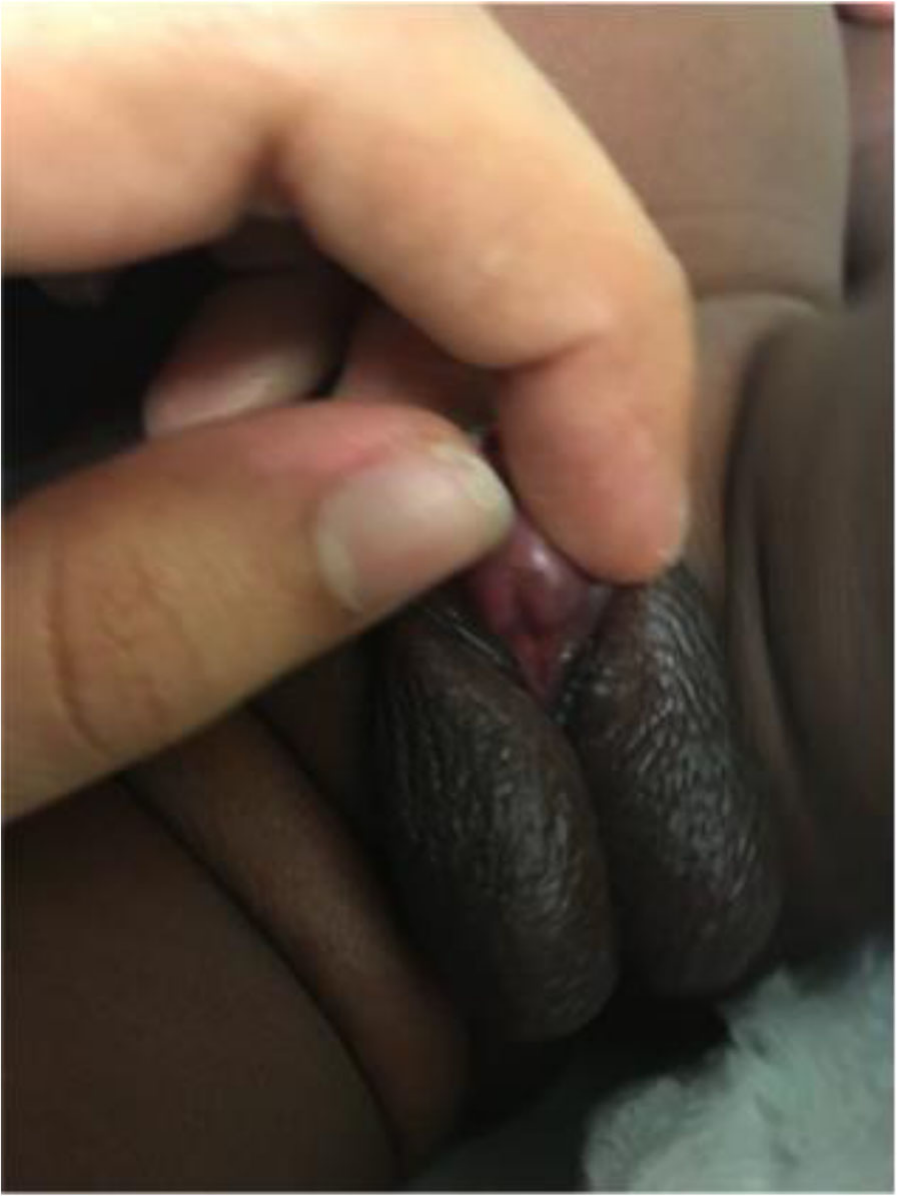
Genitalia of the patient at 6 weeks of life shows marked undervirilization with micropenis, chordee, perineoscrotal hypospadias and bifid scrotum

The patient is a West African male neonate born to consanguineous parents (second cousins). The patient’s mother is an 18-year-old G2P1 from Mali and the father is a 32-year-old from Ivory Coast who has 4 healthy children from a previous relationship. All prenatal care was in the Ivory Coast (only limited prenatal history was available) and delivery in the USA. Mother had vaginal discharge starting in her 8th week of gestation for which she was prescribed “Polygynax” vaginal capsules composed of nystatin-100,000 International Units (IU), neomycin sulfate-35,000 IU and polymyxin B-35,000 IU. She used approximately 60 capsules of Polygynax from the 8th week of gestation until delivery. The mother received betamethasone (12 mg) intramuscularly 11 h prior to delivery. The patient was born at 31 weeks of gestation by vaginal delivery with an appropriate gestational weight of 1.4 kg. His care for the first 5 weeks was at another institution. The patient required nasal continuous positive airway pressure for the first 5 days of life for respiratory distress syndrome. He also had hyperbilirubinemia requiring phototherapy. He was assigned male sex at birth. The genitalia were markedly undervirilized with a 1 cm × 0.7 cm phallus, chordee, perineo-scrotal hypospadias and bifid scrotum with bilaterally palpable gonads (Fig. 1). Serum 17-OHP on first DOL was 516 ng/dl (normal < 360 ng/dl), 24 h after prenatal administration of 12 mg betamethasone. Newborn screen on DOL 3 was positive for congenital adrenal hyperplasia with elevated 17-OHP of 304 ng/ml (normal < 35 ng/ml). Pelvic ultrasound showed no Mullerian structures. The karyotype was 46,XY and Sex-determining Region Y gene was positive. Testosterone and DHT were normal; basal serum 17-OHP, dehydroepiandrosterone sulphate (DHEAS) and 11-Deoxycortisol were slightly elevated (Table 1). All steroids were measured using liquid chromatography mass spectrometry. Cosyntropin administration (250 mcg) on DOL 3, indicated an excessive elevation of 17-OHP and 17- Hydroxypregnenolone and an acceptable (but not robust) cortisol response for the baby’s birth weight and prematurity (Table 1). Not all adrenal steroids were measured due to baby’s size. The patient was started on hydrocortisone (15 mg/m^2^) on DOL 4 and fludrocortisone (0.1 mg daily) and sodium chloride (NaCl) (1 g daily) on DOL 12 (serum sodium 134 mmol/L, potassium 6.9 mmol/L and plasma renin activity [PRA] 174 ng/ml/hr). The patient’s care was transferred to us at the Kravis Children’s Hospital at Mount Sinai at 5 weeks of age while on hydrocortisone, fludrocortisone and NaCl. Physical examination at that point was notable for severe genital undervirilization with no obvious skeletal abnormalities. Genetic testing (XomeDxSlice, GeneDx) was negative for 16 genes involved in adrenal and gonadal development (*CYP21A2, HSD3β2, AR, ARX, POR, CBX2, HSD17B3, CYP11A1, MAMLD1, LHCGR, AKR1C4, AKR1C2, MAP 3 K1, WNT4, WT1,* and *ZFPM2*). A chromosomal microarray test revealed a small region of allelic homozygosity (12.2 mb) of unknown significance.

**Table 1:** Laboratory results before and during treatment with hydrocortisone, fludrocortisone and salt. (B- Baseline, S- Stimulated with 250 mcg of cortrosyn on DOL 3)

	Reference range	DOL 2	DOL 3	DOL 10	7 weeks	3.75 months	5 months	8 months
**Hydrocortisone (mg/m2/day)**				15	30	20	12	11
**Fludrocortisone (mg)**					0.2	0.15	0.1	0.05
**NaCl (gram)**					1	2	1	1
**ACTH (pg/ml)**	7.2–63		135			15		
**Cortisol (mcg/dl)**	3–23		6.5 (B)					
			**10 (S)**					
**Sodium (mmol/L)**	134–144	142	144	134	137		138	
**Potassium (mmol/L)**	3.8–5.3	6.3	5.8	6.9	5.4			
**Bicarbonate (mmol/L)**	15–25	18	19	23	17.7		18.6	
**Plasma Renin Activity (PRA) (ng/ml/hr)**	Premature:			174.1	0.933	1.6	0.618	1.2
	1–7 Days:							
	11–167							
	1–11 Months:							
	2.35–37							
**Aldosterone (ng/dl)**	Premature:			100				
	12–736							
	1–11 months:							
	5–90							
**17-Hydroxy progesterone (ng/dl)**	Premature	516	**4853 (S)**			45		< 10
	186–472 (B)							
	334–1725 (S)							
	2 m-1 year: < 91							
**17-Hydroxy pregnenelone (ng/dl)**	Premature		**> 4000(S)**		21			
	559–2906 (B)							
	831–9760 (S)							
	6 -11 m:							
	42–540							
**Dehydroepi-androsterone Sulfate (mcg/dl)**	122–710 (B)		**2415 (B)**					
**11-Deoxycortisol (ng/ml)**	< Or = 235 (B)		**1431(B)**					
**Deoxycorticosterone (ng/dl)**	28–78 (B)		65 (B)					
**Corticosterone (ng/dl)**	201–5030 (B)		992 (B)					
**Androstenedione (ng/dl)**	1-11 m:						< 10	15
	< 10–37							
**Testosterone (ng/dl)**	31–35 weeks	383						
	Day 4							
	37–198							
**Dihydrotestosterone (ng/dl)**	Premature:		23					
	10–53							

At corrected gestational age of 2 months and 3 weeks, LH was 2.4 mIU/ml and testosterone 83.4 ng/dL, consistent with mini puberty with interval growth in penile length and width. Starting at 2 months of age, hypertension, suppressed PRA, low-normal Adrenocorticotrophic hormone (ACTH) and 17-OHP levels were documented and medications were gradually decreased and discontinued at 8 months of age (Table 1). Three days after discontinuing treatment, serum cortisol was measured before and 60 min after administration of Cosyntropin (250 mcg) and serum testosterone and DHT were measured before and on the 4th day after intramuscular administration of 15,000 IU of hCG divided over 3 days. Adrenal and testicular responses to stimulation were normal (Table 2). Patient underwent hypospadias repair and chordee release at 10 months of age. At 15 months of age, penile measurement was 3 cm × 1.5 cm and he was doing well without medications.

**Table 2:** Adrenal and testicular function after cortrosyn and hCG stimulation obtained at 8 months

**CORTROSYN STIMULATION TEST (250 mcg)**	**Reference value**	**Baseline**	**Stimulated (1 h)**
**ACTH (pg/ml)**	6–48	11	
**Cortisol (mcg/dl)**	2.8–23	19	29
**Aldosterone (ng/dl)**	5–90	38	
**Renin (ng/ml/hr)**	2–37	7.8	
**17-Hydroxypregnenolone (ng/dl)**	42–540	528	1150
**17-Hydroxyprogesterone (ng/dl)**	< 91	57	182
**Androstenedione (ng/dl)**	< 10–37	< 10	< 10
**DHEA (ng/dl)**	< 136	78	114
**Testosterone (ng/dl)**	< 2.5–10	3.6	3.8
**Dihydrotestosterone (ng/dl)**	< 3	1.4	
**Corticosterone (ng/dl)**	80–1500	871	
**Deoxycorticosterone (ng/dl)**	7–49	15	43
**11-Deoxycortisol (ng/dl)**	< 10–156	54	107
**Estrone (pg/ml)**	< 15	< 2.5	6
**Progesterone (ng/dl)**	< 10–15	< 10	52
**hCG STIMULATION TEST (15,000 IU over 3 days)**		**Baseline**	**Day 4**
**LH (mIU/ml)**	0.02–7	0.228	
**FSH (mIU/ml)**	0.16–4.1	1.3	
**Testosterone (ng/dl)**	< 2.5–10	3.6	587
**Dihydrotestosterone (ng/dl)**	< 3	1.4	111
**Androstenedione (ng/dl)**	< 10–37	< 10	15
**Estrone (pg/ml)**	< 15	< 2.5	3.8
**Estradiol (pg/ml)**	< 15	< 1	1.5

## Discussion and conclusions

The patient is a 46,XY male infant born with marked undervirilization and mild transient glucocorticoid and mineralocorticoid abnormalities, to a mother treated with prolonged high dose vaginal nystatin. The nystatin use began at 8 weeks of gestation and continued until delivery at 31 weeks; the cumulative dose was much higher than the recommended safe dose. While the standard dose of nystatin for vaginal candidiasis is one application of 100,000 IU daily for 7–14 days, the patient’s mother used a total dose of over 6 million IU [3].

The degree of undervirilization noted in our patient suggested a defect in androgen production and/or action during fetal life. The cortisol level on cosyntropin testing although not robust, could be considered appropriate for the patient’s prematurity, size and antenatal steroid treatment prior to delivery. Although the elevated baseline and stimulated 17-OHP can be explained by prematurity, we have no explanation for the elevated DHEAS and 11-Deoxycortisol noted at birth which raised the possibility of mild, transient abnormalities in cytochrome P450 and *CYP11B1* activities; genetic testing done at 5 months of life was however negative for all adrenal defects. Moreover, in 46,XY patients these enzymatic abnormalities do not cause undervirilization.

The initial 17-OHP level of 516 ng/dl (normal < 360 ng/dl) on DOL 1 may have been attenuated by the betamethasone administration 11 h prior to delivery. Due to restrictions with blood drawing, DHEA/Androstenedione ratio was not determined but the normal testosterone and DHT at birth made *3β-HSD* deficiency unlikely; more so genetic testing was normal. The possibility of Cytochrome P450 Oxidoreductase (*POR*) deficiency was considered but the lack of associated skeletal abnormalities made it unlikely. Although microarray testing did not show any defects in the genes associated with adrenal or gonadal development, certain rare genetic abnormalities cannot be fully excluded. The possibility of a post zygotic mutation leading to mild androgen insensitivity cannot be 100% excluded. The evidence of normal mini puberty with interval phallic growth and the normal androgen receptor (*AR*) gene testing argue against androgen insensitivity as the cause of our patient’s severe undervirilization. The possibility of 5-alpha reductase *(SRD5A2*) deficiency was considered but was ruled out by the normal baseline and hCG stimulated testosterone / DHT ratio. Although no testing was done for a *SF-1* mutation, the transient adrenal abnormalities, normal gonadotropins and testosterone levels, and appearance of the genitalia argue against this genetic mutation. The transient nature of P450 steroidogenesis disruption in adrenals and gonads as well as the normal T/DHT ratio strongly suggest that our patient’s undervirilization was due to factors other than genetic causes. We realize that although the patient’s initial adrenal steroid results were somewhat abnormal, they were not consistent with any type of congenital adrenal hyperplasia. Although congenital anatomic genital abnormalities are usually associated with other anatomic developmental defects (abdominal wall defect, cloacal exstrophy, renal anomaly) the possibility of an isolated genital abnormality cannot be completely ruled out. The normal response to cortrosyn after discontinuation of all medications support the transient nature of adrenal steroid abnormalities. Having received the baby on treatment and given his prematurity and size, we were not aggressive in weaning the medications and only completely discontinued them at 8 months.

We postulate that prolonged maternal treatment with high dose nystatin may have led to fetal exposure and interfered with the fetal production and/or action of testosterone and DHT only during the critical period of sexual differentiation along with mild disruption in some adrenal corticosteroid pathways still present at birth.

The abnormalities in our patient are similar to those reported after ketoconazole use; while ketoconazole inhibits fungal P450 14α-lanosterol demethylase preventing ergosterol synthesis, nystatin binds directly to ergosterol creating fungal cell wall pores leading to cell death [4, 5]. In humans, ketoconazole inhibits multiple adrenal and gonadal P450 enzymes and binds to the androgen receptor interfering with DHT action [1, 6]. Cholesterol is the essential precursor of steroidogenesis in adrenals and gonads. Evidence of adrenal insufficiency and genital ambiguity in Smith-Lemli-Opitz syndrome, an inborn error of cholesterol synthesis, underscores the importance of cholesterol in steroidogenesis [7]. Nystatin was shown to bind to cholesterol in in vitro studies [8]. The clinical abnormalities seen in our patient are similar but not identical to those caused by ketoconazole. It is possible that nystatin interferes with adrenal or gonadal steroidogenesis by an as yet unknown mechanism.

Population based case control studies indicated that the use of nystatin at 1.5–3 million IU orally for 3–6 days during the 3rd and 4th months of gestation was associated with hypospadias [2, 9].

While in most of these reports, infants were exposed to a single course of nystatin only during the first trimester; our patient was exposed to more than four times the recommended dose throughout pregnancy. Administration of nystatin in animals has been shown to cause oligospermia [10].

In addition to nystatin, Polygynax contains neomycin sulphate and polymyxin B. To our knowledge neomycin was not reported to cause any untoward effects on adrenal/gonadal steroidogenesis or to be associated with hypospadias. An in vitro study reported that polymyxin B acts as a partial ACTH agonist; this however does not explain the findings in our patient [11]. Thus, it is possible that the prolonged and excessive maternal use of nystatin and subsequent fetal exposure may have caused the abnormalities in our patient.

We report severe fetal undervirilization and multiple steroidogenenic abnormalities in a 46,XY infant. All hormonal abnormalities were transient. It is of note that the androgen pathway, although severely affected in utero, had completely recovered at birth, while the corticoid pathway required a longer time to normalize. Based on the findings in our patient, literature reports of hypospadias in infants exposed in utero to nystatin and the known untoward effects of antifungals on steroidogenesis, we suggest that nystatin should be evaluated further for possible adrenal and androgen synthesis disruption.

## Data Availability

Not applicable.

## Abbreviations

DHT: Dihydrotestosterone
IU: International units
17-OHP: 17-Hydroxyprogesterone
DOL: Day of life
DHEAS: Dehydroepiandrosterone sulphate
NaCl: Sodium chloride
PRA: Plasma renin activity
ACTH: Adrenocorticotrophic hormone
hCG: Human chorionic gonadotropin
CYP21A2: CAH 21 hydroxylase
HSD3β2: 3β-hydroxysteroid dehydrogenases
AR: Androgen receptor
ARX: Aristaless related homeobox
POR: Cytochrome P450 Oxidoreductase
CBX2: Chromobox protein homolog 2
HSD17B3: Hydroxysteroid 17 beta dehydrogenase 3
CYP11A1: Cytochrome P450 family 11
MAMLD1: Mastermind like domain containing protein 1
LHCGR: Luteinizing hormone/Choriogonadotropin receptor
AKR1C4: Aldoketo reductase family 1 member C4
AKR1C2: Aldoketo reductase family 1 member C2
MAP 3 K1: Mitogen activated protein kinase 1
WNT4: Wingless type MMTV integration site family member 4
WT1: Wilms tumor 1
ZFPM2: Zinc finger protein, multitype 2

## References

[1] Eil C (1992). Ketoconazole binds to the human androgen receptor. Horm Metab Res.

[2] Czeizel AE, Kazy Z, Puho E (2003). A population-based case-control teratological study of oral nystatin treatment during pregnancy. Scand J Infect Dis.

[3] Pappas PG, Rex JH, Sobel JD, Filler SG, Dismukes WE, Walsh TJ (2004). Guidelines for treatment of candidiasis. Clin Infect Dis.

[4] Awanish Kumar Ph. D AJ (2017). Anticandidal agents: Academic Press.

[5] Loose DS, Kan PB, Hirst MA, Marcus RA, Feldman D (1983). Ketoconazole blocks adrenal steroidogenesis by inhibiting cytochrome P450-dependent enzymes. J Clin Invest.

[6] Sonino N (1987). The use of ketoconazole as an inhibitor of steroid production. N Engl J Med.

[7] Donoghue SE, Pitt JJ, Boneh A, White SM (2018). Smith-Lemli-Opitz syndrome: clinical and biochemical correlates. J Pediatr Endocrinol Metab.

[8] Recamier KS, Hernandez-Gomez A, Gonzalez-Damian J, Ortega-Blake I (2010). Effect of membrane structure on the action of polyenes: I. Nystatin action in cholesterol- and ergosterol-containing membranes. J Membr Biol.

[9] Mavrogenis S, Urban R, Czeizel AE, Acs N (2014). Maternal risk factors in the origin of isolated hypospadias: a population-based case-control study. Congenit Anom (Kyoto).

[10] Foote RH (2002). Spermicidal effects of amphotericin B and nystatin on bull and rabbit sperm and contraceptive effects in rabbits. Contraception..

[11] Widmaier EP, Iida S, Hall PF (1987). The effect of polymyxin B on steroidogenesis from adrenocortical cells. Endocrinology..

